# Comparative and Phylogenetic Analysis of the Complete Chloroplast Genomes of *Lithocarpus* Species (Fagaceae) in South China

**DOI:** 10.3390/genes16060616

**Published:** 2025-05-22

**Authors:** Shi Shi, Ziyan Zhang, Xinhao Lin, Linjing Lu, Keyi Fu, Miaoxin He, Shiou Yih Lee, Hui Yin, Jingwei Yu

**Affiliations:** 1 Guangdong Provincial Key Laboratory of Pharmaceutical Bioactive Substances, Guangdong Pharmaceutical University, Guangzhou 510006, China; shis@scau.edu.cn (S.S.);; 2 South China Limestone Plants Research Center, College of Forestry and Landscape Architecture, South China Agricultural University, Guangzhou 510642, China; 3 Faculty of Health and Life Sciences, INTI International University, Nilai 71800, Malaysia; 4 Faculty of Nursing, Shinawatra University, Pathum Thani 12160, Thailand; 5 School of Basic Medical Sciences, Guangdong Pharmaceutical University, Guangzhou 510006, China

**Keywords:** chloroplast genomes, comparative genomics, genetic resources, *Lithocarpus*, phylogenetics analysis

## Abstract

**Background/Objectives**: In South China, *Lithocarpus* species dominate mixed evergreen broadleaf forests, forming symbiotic relationships with ectomycorrhizal fungi and serving as food resources for diverse fauna, including frugivorous birds and mammals. The limited understanding of chloroplast genomes in this genus restricts our insights into its species diversity. This study investigates the chloroplast genome (cp genome) sequences from seven *Lithocarpus* species, aims to elucidate their structural variation, evolutionary relationships, and functional gene content to provide effective support for future genetic conservation and breeding efforts. **Methods**: We isolated total DNA from fresh leaves and sequenced the complete cp genomes of these samples. To develop a genomic resource and clarify the evolutionary relationships within *Lithocarpus* species, comparative chloroplast genome studies and phylogenetic investigations were performed. **Results**: All studied species exhibited a conserved quadripartite chloroplast genome structure, with sizes ranging from 161,495 to 163,880 bp. Genome annotation revealed 130 functional genes and a GC content of 36.72–37.76%. Codon usage analysis showed a predominance of leucine-encoding codons. Our analysis identified 322 simple sequence repeats (SSRs), which were predominantly palindromic in structure (82.3%). All eight species exhibited the same 19 SSR categories in similar proportions. Eight highly variable regions (ndhF, ycf1, trnS-trnG-exon1, trnk(exon1)-rps16(exon2), rps16(exon2), rbcL-accD, and ccsA-ndh) have been identified, which could be valuable as molecular markers in future studies on the population genetics and phylogeography of this genus. The phylogeny tree provided critical insights into the evolutionary trajectory of Fagaceae, suggesting that *Lithocarpus* was strongly supported as monophyletic, while Quercus was inferred to be polyphyletic, showing a significant cytonuclear discrepancy. **Conclusions**: We characterized and compared the chloroplast genome features across eight *Lithocarpus* species, followed by comprehensive phylogenetic analyses. These findings provide critical insights for resolving taxonomic uncertainties and advancing systematic research in this genus.

## 1. Introduction

As the second-largest genus in *Fagaceae*, *Lithocarpus* comprises approximately 300 to 350 species found across various regions globally [1]. This genus is a critical component of montane and lowland ecosystems, where it plays a crucial role in shaping Northern Hemisphere forests, including temperate, subtropical, and tropical ecosystems [2,3]. Members such as *Quercus* (oaks), *Castanopsis* (chinquapins), and *Lithocarpus* (stone oaks) are dominant species in their habitats, reflecting their ecological adaptability and evolutionary success [4]. Among these, *Lithocarpus* is a key component of subtropical evergreen broad-leaved forests, yet its genomic background and evolutionary history remain uncleared compared to temperate relatives like *Quercus* and Fagus [5,6,7]. Beyond their ecological roles, several species hold significant economic value. For example, *Lithocarpus litseifolius* leaves are used to produce “sweet tea”, a traditional beverage in south China, which has both medicinal and edible functions, exhibit potent antioxidant and anticancer activities [8]. Similarly, extracts from *Lithocarpus polystachyus* rich in dihydrochalcones with demonstrated antidiabetic and anti-inflammatory properties, underscoring the genus’s potential in ethnopharmacology and modern drug discovery [9,10]. Despite their ecological and economic significance, the taxonomy of this genus remains challenging. Traditional identification based on morphological traits is not only unreliable but also time-consuming. In some cases, there are no obvious phenotypic differences among species, while morphological characteristics often show considerable intraspecific variations. Thus, the phylogenetic relationship of *Lithocarpus* still needs to be resolved.

Recent advances in molecular systematics and high-throughput sequencing technologies have significantly improved our understanding of the phylogeny of *Lithocarpus* [11,12]. Traditional morphological classifications have been challenged by the complex phenotypic variation within the genus, whereas molecular data have provided new insights into its evolutionary history. Studies have consistently supported the monophyly of *Lithocarpus* using plastid genes and nuclear markers; however, significant genetic heterogeneity observed in some widespread or ecologically diverse species suggests potential cryptic speciation or hybrid introgression events [11,12,13,14].

As key photosynthetic organelles, chloroplasts (cp) harbor circular DNA molecules (cpDNA) that exhibit haploid characteristics and are predominantly maternally transmitted in angiosperms [15]. Chloroplast genome sequencing has revolutionized plant systematics and evolutionary biology by providing a robust molecular framework to resolve long-standing taxonomic ambiguities [16,17,18]. Unlike nuclear genomes, cpDNA exhibit a conserved quadripartite structure (large single-copy [LSC], small single-copy [SSC], and inverted repeat [IR] regions), low recombination rates, and maternal inheritance, making them ideal markers for reconstructing deep evolutionary divergences and recent radiations [19,20]. Comparisons with cpDNA can reveal lineage-specific structural variations, such as IR expansions/contractions, gene loss and inversions, which often correlate with ecological adaptations or historical biogeographic events. For instance, in Fagaceae, cpDNA analyses have clarified the polyphyletic nature of *Castanopsis* and *Lithocarpus*, revealing complex speciation driven by Pleistocene climatic fluctuations [21]. Moreover, codon usage bias analyses and selective pressure assessments (e.g., dN/dS ratios) in chloroplast genes (e.g., rbcL, matK, ndhF) have provided insights into adaptive evolution, particularly in response to light availability and temperature gradients [22]. These advancements highlight the dual utility of cpDNA as both phylogenetic anchors for resolving species boundaries and functional genomic tools for detecting the molecular basis of ecological diversification.

In this study, we sequenced and analyzed the complete chloroplast genomes of eight ecologically divergent *Lithocarpus* species endemic to South China: *Lithocarpus uvarifolius*, *L. crassifolius*, *L. corneus*, *L. calophyllus*, *L. oleifolius*, *L. longipedicellatus*, *L. litseifolius*, and *L. longanoides*. Subsequently, we conducted a comparative analysis of these chloroplast (cp) genomes to examine their sequence architecture and genetic variations. We analyzed codon usage bias and identified hotspots of high nucleotide diversity within the cp genomes. Additionally, to elucidate evolutionary relationships among *Lithocarpus* species, we generated a phylogenetic tree using complete chloroplast genomes from 76 taxa. The *Lithocarpus* species employed in this study are widely distributed across southern China, predominantly occupying low-altitude regions with similar ecological niches. Through these investigations, our study aims to elucidate the molecular-level variations among these species, deepen our understanding of their evolutionary relationships, and provide a foundation for the development and utilization of these plant resources.

## 2. Materials and Methods

### 2.1. Collection and Preservation of Materials

The fresh leaves of six *Lithocarpus* species (*L. uvarifolius*, *L. crassifolius*, *L. corneus*, *L. calophyllus*, *L. longanoides*, *L. oleifolius*, and *L. longipedicellatus*) were collected in July 2023 from natural populations in the Heishiding Mountain (23°27′56.30″ N, 111°54′19.95″ E), Guangdong Province, China, while *L. longipedicellatus* was collected in August 2024 from Jianfengling Mountain (18°44′24.50″ N, 108°51′39.94″ E), Hainan Province, China. All seven specimens were collected across representative microhabitats within the reserve to ensure the coverage of genetic variation. All collected specimens underwent immediate dehydration with silica gel followed by ultra-low temperature preservation (−80 °C). The voucher specimens were deposited in the Herbarium of South China Agricultural University (CANT) (Accession number: 32,213~32,220).

### 2.2. Chloroplast Genome Assembly and Comparative Analysis

#### 2.2.1. DNA Extraction and Sequencing

The total DNA was extracted from the seven *Lithocarpus* species using a modified cetyltrimethylammonium bromide (CTAB) method. DNA integrity was assessed via 1% agarose gel electrophoresis, and concentrations were quantified using a Nanodrop 2000 spectrophotometer (Thermo Scientific, Shanghai, China). Sequencing libraries were prepared with the NEBNext^®^ Ultra™ DNA Library Prep Kit for Illumina (NEB, Ipswich, MA, USA; Catalog #E7370L), targeting an average fragment size of 350 bp. Paired-end sequencing (PE 150) was performed on the Illumina Novaseq Xplus platform, yielding about 6 GB of raw data per sample.

#### 2.2.2. Chloroplast Genome Assembly and Annotation

Raw reads were preprocessed with Fastp v0.23.4 to remove adapters [23], trim low-quality bases (Q < 20), and discard reads with above 10% N content and the length shorter than 140 nt. Chloroplast genomes were assembled using GetOrganelle v1.7.7.1. [24], utilizing the cp genome of *L. longipedicellatus* as the reference. The assembled chloroplast genome was annotated using online annotation software Geseq (version 2.03) (https://chlorobox.mpimp-golm.mpg.de/geseq.html, accessed on 16 March 2025) and CPGAVAS2 (Last updated at 2019) (http://47.96.249.172:16019/analyzer/annotate, accessed on 16 March 2025) [25,26]. These tools were employed to determine the start positions of the chloroplast genome and the IR regions, as well as to annotate the genes present. Finally, manual adjustments were employed to ensure the accuracy of the annotations and to identify any potential errors.

#### 2.2.3. Chloroplast Genomes Comparison

Considering that *L. litseifolius* is morphologically similar to the *Lithocarpus* species we collected and their distribution ranges overlap, we downloaded the complete chloroplast genome (NC_063927.1) of this species for comparative analysis. mVISTA is a set of programs used to compare the DNA sequences of millions of base pairs in length between two or more species, and to visualize these comparison results in conjunction with annotation information. Using the online tool mVISTA (Last updated at 2021) (https://genome.lbl.gov/vista/mvista/submit.shtml, accessed on 16 March 2025), we upload the aligned fasta file and each sample’s separate annotation file at the same time for analysis [27]. Nucleotide polymorphism (Pi) is a parameter that measures the level of polymorphism in a specific population, which refers to the average difference in nucleotides at each position between two randomly selected DNA sequences within the same population. This analysis uses MEGA X for alignment and Dnasp5 (version 5.10.1) software to calculate all sequences, with the window set to 400 bp and the step set to 200 bp [28,29].

#### 2.2.4. IR Boundary Variation Analysis

The inverted repeat (IR) regions of chloroplast genomes are considered the most conserved regions; however, their boundary sequences can exhibit dynamic changes, including outward expansion or inward contraction. These variations can lead to alterations in the copy number of associated genes or the generation of pseudogenes in the boundary regions. Such phenomena are common during chloroplast genome evolution and are a primary cause of length variation among chloroplast genomes. In this study, we utilized the online software IRscope (Last updated at 2018) (https://irscope.shinyapps.io/irapp/, accessed on 16 March 2025) to analyze the genetic structure near the junctions connecting the IR regions with the short single-copy (SSC) and long single-copy (LSC) regions [30]. The analysis was performed by uploading the GenBank-annotated files of eight *Lithocarpus* species. This approach allowed us to visualize and compare the structural dynamics at the IR boundaries across the eight species, providing insights into the evolutionary mechanisms driving the divergence of chloroplast genome architecture within the genus *Lithocarpus*.

#### 2.2.5. Repetitive Sequence Analysis

Repetitive sequences are known to play a crucial role in genomic rearrangements and recombination, and they may also exhibit phylogenetic information within certain populations. In this study, we employed the REPuter (version 2.5.18) software (https://bibiserv.cebitec.uni-bielefeld.de/reputer, accessed on 16 March 2025) to quantify the number of long sequence repetitive fragments in each sample [31]. The REPuter software operates on the principle of detecting repetitive sequences within a genome using algorithms. It can identify various types of repeats, including forward (f), reverse (r), complement (c), and palindromic (p) repeats. For our analysis, we set the Hamming Distance to 3, the Maximum Computed Repeats to 50, and the Minimal Repeat Size to 30. Meanwhile, we utilized the MISA (microsatellite identification tool) online platform [8] to analyze various data related to SSRs, including the number of repetitions, repeat units, and lengths. The primary principle of this tool is to scan the given genomic sequences to search for repeat units within specific length ranges. These repeat units can consist of sequences of 1 to 6 nucleotides, such as mononucleotides (e.g., repetitions of A, T, C, G) and dinucleotides (e.g., repetitions of AT, CG, etc.).

#### 2.2.6. Codon Usage Bias Analysis

The relative probability of a specific codon among its synonymous codons reflects the degree of codon bias. Relative synonymous codon usage (RSCU) values were calculated to quantify codon bias [32]. This analysis is crucial for understanding species’ evolutionary pressures and further genetic research. In this analysis, we used the DAMBE 7 software to calculate RSCU values for each sample individually. During the analysis, duplicate genes were removed, and the editing of non-ATG start codons for methionine was considered to ensure accurate results.

#### 2.2.7. Phylogenetic Tree Construction

To analyze the phylogenetic relationships within Fagaceae, we conducted a plastid genome phylogenomic study using 71 complete chloroplast genomes. The dataset including 11 *Castanopsis* species, 28 *Quercus* species, 25 *Lithocarpus* plastomes, and 5 *Castanea* species, with *Carpinus laxiflora* and *Morella salicifolia* set as the outgroup. Seven plastomes of *Lithocarpus* were newly sequenced in this study, and another sixty-eight were sourced from NCBI (accession numbers of the complete chloroplast genomes are pro-vided in Supplementary Table S1).

Sequence alignment was performed using MEGA X with default parameters [33]. The optimal nucleotide substitution model (K3Pu + F + I + G4) was selected using ModelFinder. The phylogenetic relationships were inferred using maximum likelihood estimation implemented in IQ-TREE (version 2.4.0), employing 1000 iterations of ultrafast bootstrap resampling with standard parameters [34]. Alignments were trimmed in Geneious (version 2025.1), and phylogenies were inferred using maximum likelihood (ML) [35]. Trees were visualized and edited in FigTree v1.4.3 [36].

## 3. Results

### 3.1. Chloroplast Genome Structure Characteristics

**Figure 1:**
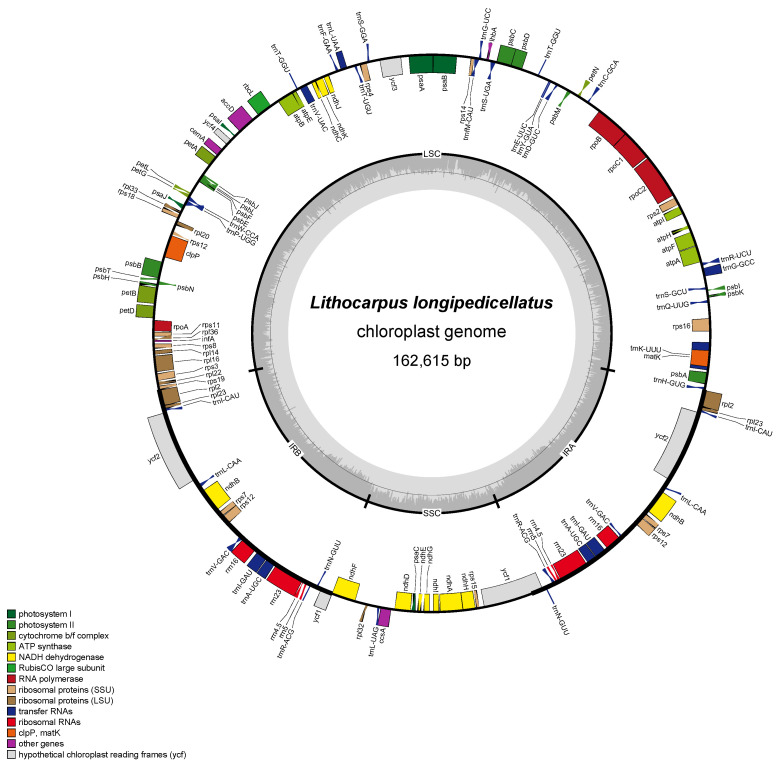
Genome map of *L. longipedicellatus* presents a typical chloroplast genome structure and content Genes illustrated within the circular diagram exhibit clockwise transcription, while those positioned externally transcribe in the counterclockwise direction. Functional gene classification is visually represented through a color-coded system.

In this study, we sequenced and assembled the complete chloroplast genome of eight *Lithocarpus* species (*L*. *uvarifolius*, *L*. *crassifolius*, *L. corneus*, *L*. *calophyllus*, *L*. *oleifolius*, *L*. *longipedicellatus*, *L*. *litseifolius*, and *L*. *longanoides*), with raw sequencing data deposited in the NCBI GenBank database (Table 1). The lengths of the nucleotide sequences of eight species ranged from 162,615 bp in *L*. *longipedicellatus* to 161,212 bp in *L*. *oleifolius*. There are four distinct regions in the chloroplast genomes of each of the eight species studied: a large single copy (LSC), a small single copy (SSC), and two inverted repeats (IRa and IRb). A representative circular chloroplast genome structure of L. longipedicellatus is shown in Figure 1. All eight species displayed similar gene content, with 130 functional genes identified: 86 protein-coding genes and 36 transfer RNA (tRNA) genes. The GC content showed minimal variation across species (36.72–36.76%), further supporting structural conservation (Table 2).

### 3.2. Comparative Analysis of Chloroplast Genomes

**Figure 2:**
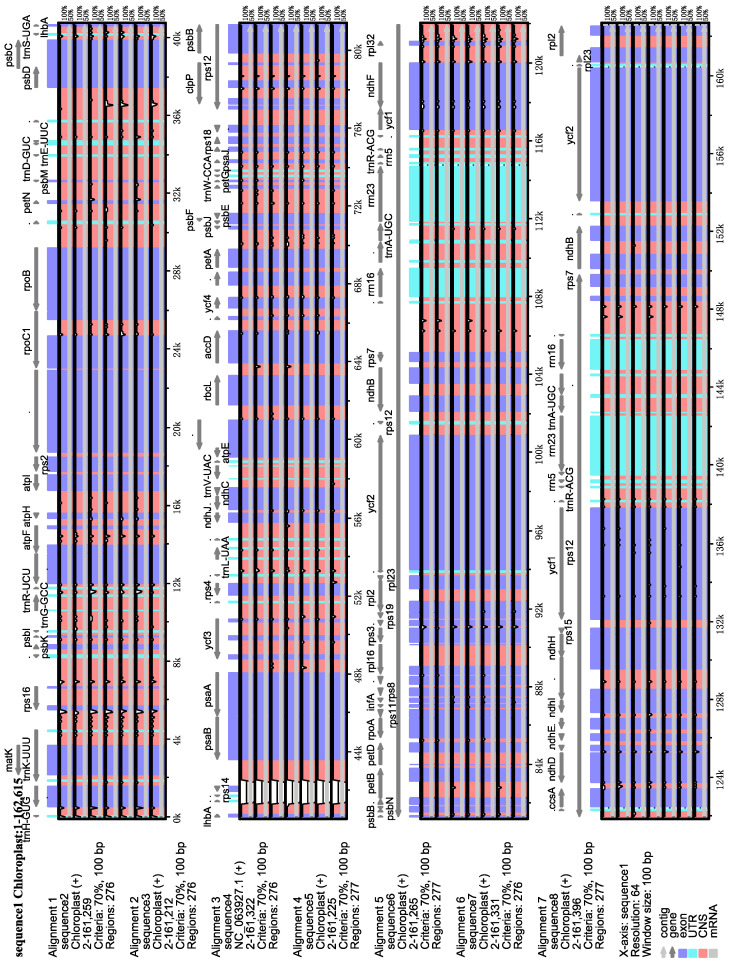
Subsequent sequence identity plots depict the chloroplast genome sequences of 8 *Lithocarpus* species. Grey directional arrows mark gene orientation. Coding elements are color-coded (purple: exons; blue: introns) with non-coding regions in red. The *y* axis shows sequence similarity (50–100%).

To accurately estimate sequence variation, we conducted multiple comparative analyses of the chloroplast genomes of the eight *Lithocarpus* species previously mentioned. The analysis revealed limited sequence variation in the chloroplast genomes of the eight species, though non-coding regions exhibited greater polymorphism than coding regions. (Figure 2).

**Figure 3:**
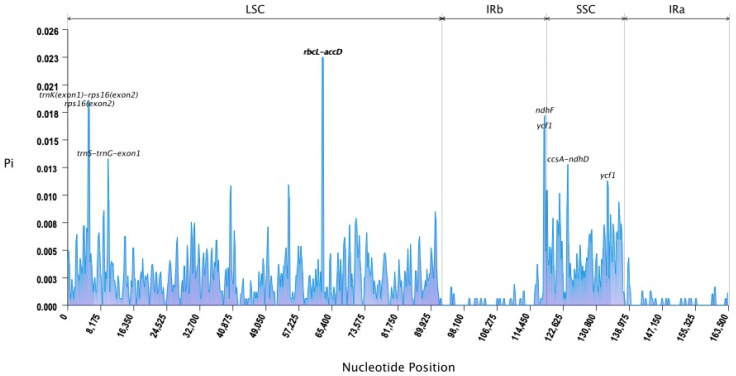
Nucleotide polymorphisms (Pi) of the 8 *Lithocarpus* chloroplast genomes. The nucleotide diversity values are plotted on the *y* axis, with the corresponding genomic positions of the sliding windows displayed along the *x* axis.

Nucleotide diversity (Pi) serves as a population genetic metric that quantifies sequence variation within a population, calculated as the average number of nucleotide differences per site between any two randomly chosen sequences from the population. Nucleotide polymorphism (Pi) can reveal the extent of nucleic acid sequence variation among different species, and regions with higher variation may provide potential molecular markers for population genetics research. We evaluated the nucleotide polymorphism in the eight chloroplast genomes to determine mutational hotspots more precisely. Using a 400 bp sliding window, we estimated Pi values, which varied between 0 and 0.02312. The top 5% of windows with the highest Pi values were selected, with a minimum threshold of 0.01161. This approach revealed five highly polymorphic regions (Pi > 0.01161), which were designated as potential mutational hotspots for molecular marker development (Figure 3). Eight regions of the chloroplast genes and gene spacer regions of the eight *Lithocarpus* species were identified as highly variable in the LSC region (Pi > 0.01161), namely *ndhF* (0.01768), *ycf1* (0.01625), *ycf1* (0.01161), *trnS-trnG-exon1* (0.01366), *trnk (exon1)-rps16 (exon2)* (0.01911), *rps16 (exon2)* (0.0183), *rbcL-accD* (0.02312), and *ccsA-ndhD* (0.1312).

### 3.3. IR Region Contraction and Expansion (Analysis of IR Boundary Variation)

**Figure 4:**
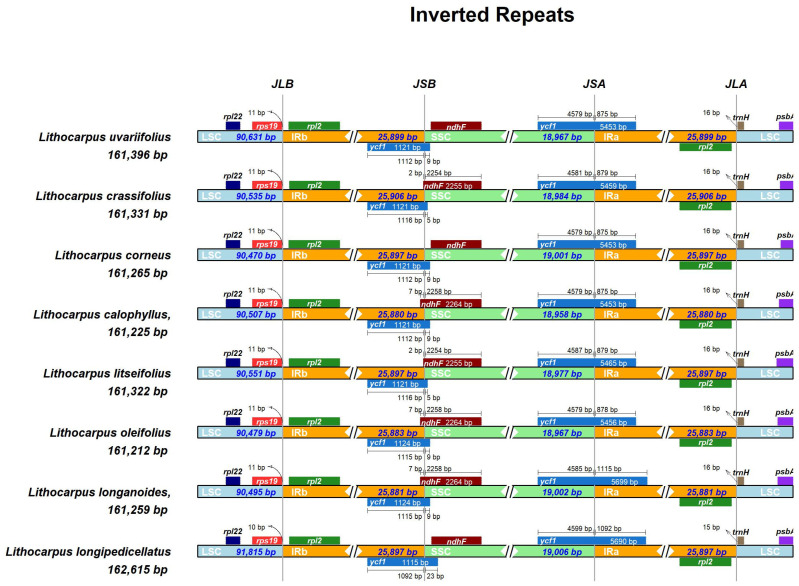
Comparison of the boundaries of a large single copy (LSC), small single copy (SSC), and inverted repeat (IR) regions in the *Lithocarpus* cpDNAs. Note: Genes are illustrated as horizontal bars in schematic diagrams, and the intervals and boundaries between genes are represented by base pair length. Structural extensions such as exon elongations or regulatory regions should be clearly marked above the corresponding bars.

The regulation of the inverted repeat (IR) region size through contraction and expansion processes is fundamental to the dynamics of chloroplast genome architecture and serves as the primary factor influencing its size variability. A comparative analysis of the chloroplast genomes of eight *Lithocarpus* species revealed size variations ranging from 161,212 bp (*L. oleifolius)* to 162,615 bp (*L. longipedicellatus*), with the latter exhibiting the largest IR region (51,794 bp). Structural alignment identified species-specific shifts in the IR/SSC junction positions, suggesting potential adaptive divergence in these regions during evolutionary diversification. Using IRscope technology, we meticulously documented the dynamic changes in chloroplast genome sizes across the eight *Lithocarpus* species (Figure 4). The analysis indicated that the IR region lengths were relatively conserved, showing no significant contraction or expansion. In contrast, the SSC (small single-copy) and LSC (large single-copy) regions exhibited length variations ranging from 17 to 161 bp, with most differences limited to a few base pairs.

Our analysis discovered that five genes—*rps19*, *rpl2*, *ndhF*, *ycf1*, and *trnH*—located nearby the LSC-IRb, SSC-IRb, SSC-IRa, and LSC-IRa boundaries. Notably, rpl19 and trnH were entirely confined to the LSC region, positioned adjacent to the LSC-IRb and LSC-IRa boundaries without crossing into the IR regions. The *ndhF* gene, mainly located in the SSC region, was found to span the IRb-SSC boundary in *L. crassifolius*, *L. litseifolius*, *L. calophyllus*, *L. oleifolius*, and *L. longanoides*, with a minimal extension of 2–7 base pairs extended into the IRb region. The ycf1 gene was identified to span both the IRb-SSC and SSC-IRa boundaries among all eight *Lithocarpus* species. At the IRb-SSC boundary, this gene predominantly resided within the IRb region, exhibiting minimal extension (5–23 bp) into the SSC region. In contrast, a more substantial genomic span was observed at the SSC-IRa boundary. The SSC-region segment of ycf1 demonstrated high sequence conservation, maintaining a consistent length ranging from 4579 to 4599 bp. However, its IRa-region counterpart displayed marked length variation, spanning 875–1115 bp.

### 3.4. Repeat Analysis

**Figure 5:**
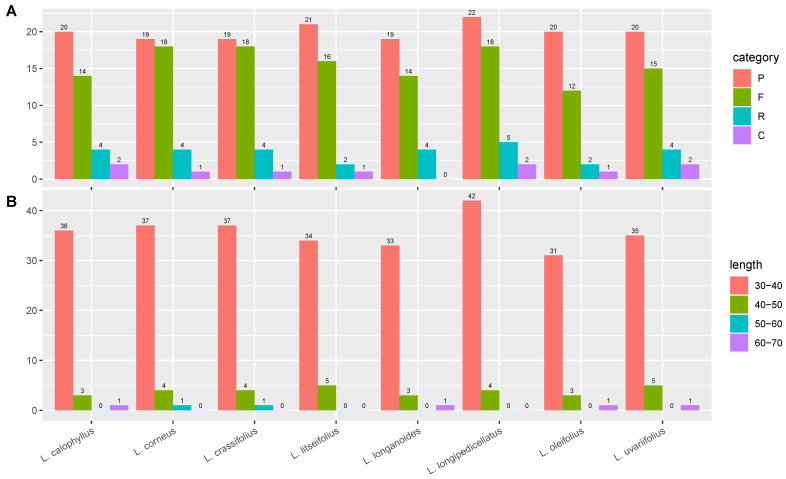
Quantitative analysis of long repeat sequences in eight *Lithocarpus* species. (**A**) Proportion of four types of long repeat sequences in each species: p represents palindromic sequences, F represents forward repeat sequences, R represents reverse repeat sequences, and C represents complementary sequences. (**B**) Proportion of repeat sequences of different lengths in each species.

Simple sequence repeats (SSRs) are abundant, highly polymorphic, uniformly distributed across the entire genome, co-dominantly inherited, and easy to detect, making them the second-generation molecular markers widely used in genetic map construction, target gene localization, genetic diversity research, molecular assisted breeding, and germplasm resource identification. Previous studies have emphasized the presence of simple repetitive motifs in the chloroplast genome, which are associated with various genomic rearrangements, recombination events, and large inversions. In this study, we compared eight species of the genus *Lithocarpus* and identified a total of 19 SSRs. The distribution of SSR types in the chloroplast genome of each species was similar, ranging from 14 to 15 types. Mononucleotide repeats (A/T and C/G) accounted for the largest proportion (80.7–83.7%), followed by dinucleotide repeats (AG/CT and AT/AT) (5.2–7.9%). The longest SSR identified was a hexanucleotide repeat (AAATAT/ATATTT, AATATT/AATATT, AAAAAT/ATTTTT, AATACT/AGTATT, and AAAGAT/ATCTTT) (Figure 5).

**Figure 6:**
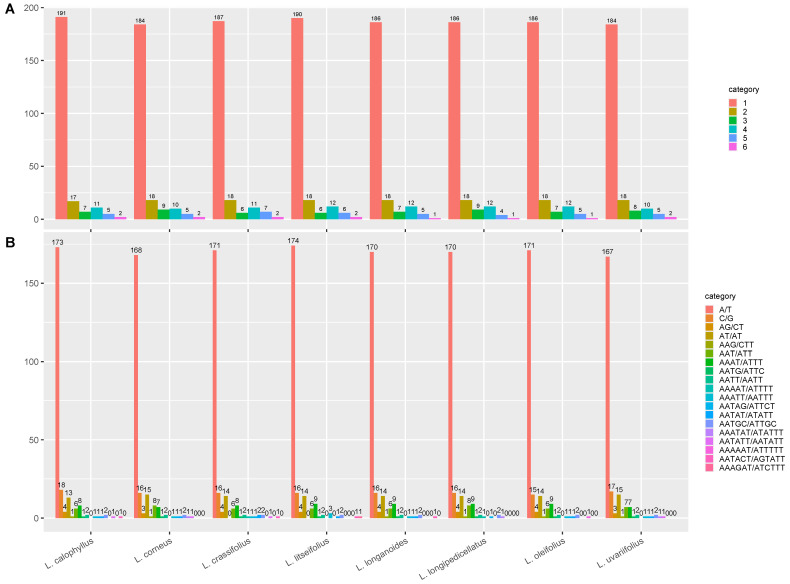
Quantitative analysis of SSRs in eight *Lithocarpus* species. (**A**) Proportion of SSRs with different repeat unit lengths in each species. (**B**) Frequency and types of identified SSRs.

Meanwhile, the type, amount, and length of the long repeat sequences were analyzed in eight *Lithocarpus* species. The analysis discovered a total of 322 significant repeat sequences, comprising 160 palindromic sequences, 125 forward repeats, 27 reverse repeats, and 10 complementary repeats. Among these investigated species, the chloroplast genome of *L. oleifolius* possesses the fewest forward repeats, with only 12 repeats identified, whereas the seven other *Lithocarpus* species exhibit significantly higher repeats numbers ranging from 14 to 18. *L. oleifolius* cpDNA also exhibits the minimal number of reverse tandem repeats, with only two repeats identified, whereas other *Lithocarpus* species display significantly higher counts ranging from 4 to 5. Notably, the quantity of palindromic repeats remains relatively conserved across species, approximately 19–22 copies, although *L. longanoides* entirely lacks complement repeats, in contrast to the 1–2 copies observed in other species (Figure 6A). The long repeat sequences were predominantly distributed within 30–40 bp, with 285 instances constituting 88.51% of the total. Notably, *L. corneus* and *L. crassifolius* exclusively exhibited repetitive elements within 50–60 bp, a distinct pattern diverging from the general trend (Figure 6B).

### 3.5. Code Usage Bias Analysis

**Figure 7:**
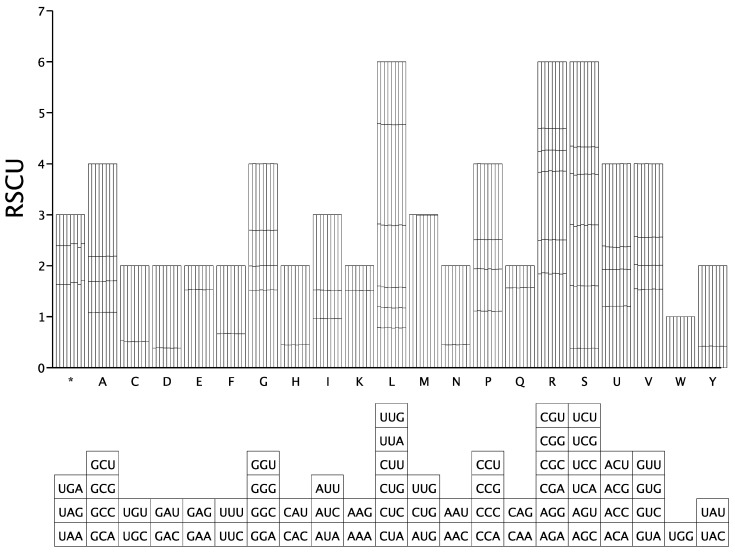
Bar chart illustrating codon usage bias across eight *Lithocarpus* species. The *x* axis denotes the 20 essential amino acids and a termination codon (*), while the *y* axis represents the codons employed for each amino acid and their corresponding usage frequencies within the analyzed dataset.

The degree of preferential codon usage can be assessed by calculating the relative frequencies of synonymous codons for each amino acid in a coding sequence. This bias can be quantified by calculating the relative synonymous codon usage (RSCU), which measures the deviation from the equal usage of synonymous codons. Investigating codon usage patterns is significantly important for elucidating species-specific evolutionary pressures and advancing genetic research. In this study, we employed DAMBE to compute RSCU values on a sample-by-sample basis, followed by the aggregation of the results. The analysis excluded redundant genes to avoid bias and accounted for non-ATG start codons encoding methionine (M) during sequence annotation. This approach ensures robustness in identifying evolutionary constraints and translational efficiency patterns across the studied samples. Our analysis revealed that the chloroplast genomes of the eight investigated *Lithocarpus* species collectively comprised 63 synonymous codons responsible for encoding 20 distinct amino acids (Figure 7). Among these amino acids, Leucine was predominantly encoded by the UUA codon, exhibiting a high RSCU value exceeding 1.90 (range: 1.9725–2.016), and Arginine (Arg) showed a preference for the AGA codon, with the RSCU value ranging from 1.8373 to 1.8584. In contrast, Tryptophan (Trp) had RSCU values precisely at 1.00. Notably, these *Lithocarpus* species exhibited similar codon usage patterns, and this similarity provides valuable reference data for further phylogenetic analysis.

### 3.6. Phylogenetic Analysis

**Figure 8:**
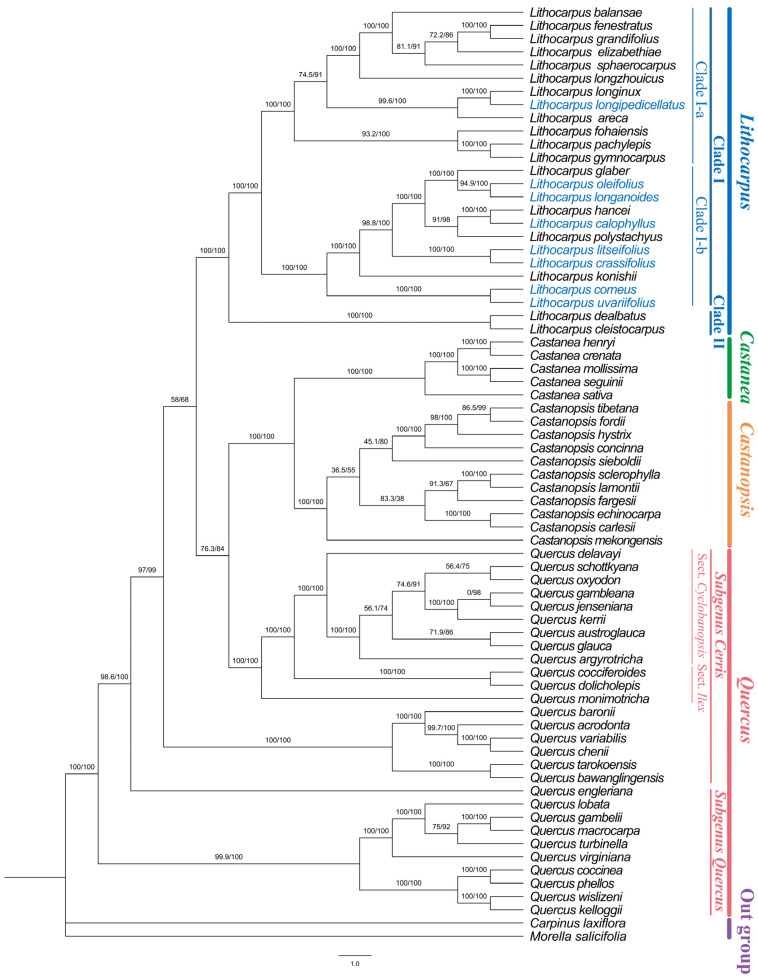
Phylogenetic analysis of chloroplast genomes from 71 *Fagaceae* species. Blue bold font indicates the eight investigated *lithocarpus* species.

Phylogenetic analysis based on maximum likelihood (ML) methods yielded a highly supported tree topology (Figure 8). All Fagaceae genera except *Quercus* demonstrated strong support for monophyly. *Castanopsis* and *Castanea* formed a well-supported sister clade (BS = 100, PP = 1.00), which clustered with the *Cerris* subgenus of *Quercus* encompassing sections *Cyclobalanopsis* and *Ilex*. *Quercus* subgenus *Quercus* occupied the most basal position in the entire Fagaceae phylogeny (BS = 100, PP = 1.00), representing the earliest-diverging lineage of the family. *Lithocarpus* was resolved into two clades (Clade I and Clade II). Clade I was further divided into two subclades: Subclade I-a, mainly consisting of species from Southwest China, included *L. longipedicellatus*, while Subclade I-b comprised species distributed in central-south China. Within clade I-b, three distinct sister–taxa pairs were identified: *L. oleifolius* and *L. longanoides*, *L. litseifolius* and *L. crassifolius*, *L. corneus* and *L. uvariifolius*. These groupings exhibited high levels of phylogenetic support (BS > 94.9, PP = 1.00). Clade II comprised *L. dealbatus* and *L. cleistocarpus* (BS = 100, PP = 1.00), forming a well-supported monophyletic group.

## 4. Discussion

The comparative analysis of chloroplast (cp) genomes in eight *Lithocarpus* species revealed critical insights into their structural conservation, evolutionary dynamics, and potential molecular markers, aligning with recent advances in cp genomics and phylogenomics. Our findings demonstrate that *Lithocarpus* cp genomes exhibit remarkable structural stability, consistent with the trends observed in other *Fagaceae* genera such as *Quercus* and *Castanopsis* [37]. This level of conservation is vital for preserving genomic stability and ensuring proper chloroplast functionality. The conserved gene content (130 functional genes) and minimal GC content variation (36.72–36.76%) across species underscore the evolutionary constraints on core photosynthetic and metabolic functions, a pattern similarly documented in stress-adapted angiosperms. Meanwhile, this conservation aligns with prior studies on *L. dealbatus* and *L. hancei*, where IR regions demonstrated a higher GC content (42.7%) than single-copy regions due to rRNA genes [38]. Notably, the IR/SSC boundary shifts in ycf1 and ndhF genes—observed in five species—may reflect adaptive divergence, as similar junction dynamics have been linked to ecological speciation in *Fagaceae* [39]. However, the quadripartite chloroplast structure (LSC/SSC/IRa/IRb) exhibited minimal length variation (161,212–162,615 bp) and near-identical GC content (36.72–36.76%), and these data suggest species-specific adaptations, potentially linked to ecological niche differentiation in *Lithocarpus*, which dominates subtropical evergreen forests under different light conditions [40,41].

Nucleotide polymorphism (Pi) is a parameter used to measure the level of polymorphism within a specific population, defined as the mean of nucleotide differences at each position between two randomly selected DNA sequences from the population. Nucleotide polymorphism (Pi) can reveal the extent of nucleic acid sequence variation among different species, and regions with higher variation may provide potential molecular markers for species identification research. In our study, five hypervariable regions (e.g., ndhF, ycf1, and rbcL-accD) with Pi values >0.01161 were identified as potential targets for molecular marker development. Notably, ycf1, which was implicated in oxidative stress tolerance and seems to be the most promising DNA barcode of land plants [42,43], exhibited dual high-Pi value (0.01625 and 0.01161), suggesting its role in adaptive divergence among *Lithocarpus* species. These results corroborate recent studies highlighting ycf1 as a hotspot for cp genome evolution in *Fagaceae* [40]. Furthermore, the rbcL-accD spacer (Pi = 0.02312) emerged as the most polymorphic region, consistently with its recognized utility in species-level phylogenetics [44]. These markers resolve taxonomic uncertainties, such as the sister relationship between *L. oleifolius* and *L. longanoides*, contradicting morphological classifications that grouped *L. oleifolius* with *L. litseifolius*. These markers might provide clues to resolve taxonomic ambiguities in *Lithocarpus*, particularly for morphologically cryptic species, which represent a persistent challenge in Fagaceae systematics.

The regulation of the inverted repeat (IR) region size through contraction and expansion processes is fundamental to the dynamics of chloroplast genome architecture and serves as the primary factor influencing its size variability. IR boundary dynamics, particularly the extension of ycf1 into the IRa region (up to 240 bp), reflect lineage-specific expansions, a phenomenon increasingly reported in Fagaceae. Such expansions may stabilize cp genome architecture against deleterious mutations, as IR regions are known to buffer structural variations. The observed ndhF overlap with IRb (2–7 bp) further supports the hypothesis that the IR boundary shifts the drive in cp genome diversification in closely related species.

Simple sequence repeats (SSRs) are abundant, highly polymorphic, uniformly distributed across the entire genome, co-dominantly inherited, and easy to detect, making them the second-generation molecular markers widely used in genetic map construction, target gene localization, genetic diversity research, molecular assisted breeding, and germplasm resource identification. Previous studies have emphasized the presence of simple repetitive motifs in the chloroplast genome, which are associated with various genomic rearrangements, recombination events, and large inversions. The predominance of mononucleotide SSRs (A/T, 80.7–83.7%) aligns with cp genome trends in angiosperm chloroplast genomes. These SSRs, coupled with abundant palindromic repeats (160/322), may facilitate genomic rearrangements and adaptive evolution. However, the functional implications of these repeats in *Lithocarpus* require experimental validation, such as assessing their correlation with phenotypic traits under environmental stress. SSR and repeat sequence analyses further highlighted evolutionary constraints. The reduced repeat numbers in *L. oleifolius* (12 forward vs. 14–18 in others) may indicate lineage-specific genomic streamlining. Codon usage bias favored UUA (Leucine, RSCU > 1.90) and AGA (Arginine), consistent with *Lithocarpus* codon adaptation patterns, potentially reflecting translational efficiency optimization.

Our maximum likelihood phylogenetic reconstruction provided critical insights into the evolutionary trajectory of Fagaceae. As shown by the phylogenetic tree, *Lithocarpus*, *Castanopsis*, and *Castanea* were resolved as monophyletic groups, consistently with previous studies [11,13]. *Quercus* has consistently been resolved as a monophyletic group in earlier phylogenetic studies based on nuclear genes [45,46,47]. Notably, in this study, the infrageneric taxa of *Quercus* based on chloroplast genomes was inferred to be polyphyletic, revealing a striking nuclear-cytoplasmic phylogenetic incongruence. This cytonuclear discrepancy aligns with findings from Zhou et al. [12], who proposed that the cooling climate of the Miocene epoch, accompanied by a sea-level drop, led to the re-emergence of land bridges (such as the Bering Strait), facilitated the interspecific hybridization of Quercus between Eurasian and North American, and ultimately drove the divergence in the genetic histories of nuclear and plastid genes.

In the basal branch of subgenus *Cerris*, species from the *Ilex* section (e.g., *Quercus tarokoensis*, *Quercus bawanglingensis*) from southern Chinese islands (Hainan and Taiwan) were nested within species from the *Cerris* section (e.g., *Quercus chenii*, *Quercus variabilis*, and *Quercus acrodonta*) widely distributed in mainland China. This pattern, consistent with Hubert et al. [45], may reflect incomplete lineage sorting or gene introgression.

This study analyzed the maternal evolutionary history of *Lithocarpus* using chloroplast genome data. A phylogenomic tree based on chloroplast genomes robustly resolved *Lithocarpus* into two major clades. One clade comprised two species from southwestern China-India and central China. Another clade included two subclades corresponding to southwestern China (subclade I-a) and central-southern China (subclade I-b), indicating a potential phylogeographic structure in the chloroplast genomes of *Lithocarpus*, consistently with previous studies [11,13].

Species collected in Guangdong in this study were clustered within subclade I-b. *Lithocarpus corneus* and *Lithocarpus uvariifolius* were clustered with high support, forming a distinct monophyletic group. These two species frequently share overlapping habitats in Guangdong, where they act as ecologically associated species, and share similar morphological traits, including deeply cupulate cupules that envelop over half of their large nuts. In Guangdong, their fruits are harvested as the shared medicinal name “Fengliu fruit” in local markets. They are distinguished by *L. corneus* having larger fruits and more elongated leaves.

Numerous studies have shown significant topological incongruence between nuclear and chloroplast genome datasets in *Lithocarpus*. This incongruence may arise from factors such as chloroplast genome convergence, introgression, incomplete lineage sorting, or differential rates of gene flow mediated by pollen and seeds [45,46,47].

## 5. Conclusions

In the present study, the complete chloroplast genomes of seven *Lithocarpus* species were sequenced and assembled, revealing a highly conserved genomic architecture with minimal variation in size (161,495–163,880 bp), GC content (36.72–37.76%), and repeat element distribution. Comparative genomic analyses with closely related species identified both shared ancestral features and genus-specific characteristics. Phylogenetic reconstruction based on these chloroplast genomes provides robust support for interspecific relationships within *Lithocarpus*. These findings significantly expand the genomic resources for Fagaceae systematics while offering new insights into the evolutionary patterns and diversification mechanisms within this ecologically important genus.

## Figures and Tables

**Table 1:** General characteristics of the 8 newly sequenced chloroplast genomes.

Species	Chloroplast Genome Size/bp	IR Length/bp	Overall GC Content %	Gene Number	tRNA Genes	Protein Coding Genes	GenBank Accession Number
*L. longipedicellatus*	162,615	25,897	36.74	130	36	86	PP234611.1
*L. longanoides*	161,259	25,881	36.75	130	36	86	PV191269
*L. oleifolius*	161,212	25,883	36.74	130	36	86	OR805597.1
*L. litseifolius*	161,322	25,897	36.73	130	36	86	NC_063927.1
*L. calophyllus*	161,225	25,880	36.73	130	36	86	PP234612.1
*L. corneus*	161,265	25,897	36.73	130	36	86	PP234614.1
*L. crassifolius*	161,331	25,906	36.72	130	36	86	PP234613.1
*L. uvariifolius*	161,396	25,899	36.76	130	36	86	PP234615.1

**Table 2:** Gene composition of the 7 newly sequenced chloroplast genomes.

Category	Gene Group	Genes
Photosynthesis	Subunits of photosystem I	*psaA, psaB, psaC, psaI, psaJ*
	Subunits of photosystem II	*psbA, psbB, psbC, psbD, psbE, psbF, psbH, psbI, psbJ, psbK, psbL, psbM, psbN, psbT*
	Subunits of NADH dehydrogenase	ndhA *, ndhB *(2), ndhC, ndhD, ndhE, ndhF, ndhG, ndhH, ndhI, ndhJ, ndhK
	Subunits of cytochrome b/f complex	petA, petB *, petD *, petG, petL, petN
	Subunits of ATP synthase	atpA, atpB, atpE, atpF *, atpH, atpI
	Large subunit of rubisco	*rbcL*
	Subunits photochlorophyllide reductase	*-*
Self-replication	Proteins of large ribosomal subunit	rpl14, rpl16 *, rpl2 *(2), rpl20, rpl22, rpl23(2), rpl32, rpl33, rpl36
	Proteins of small ribosomal subunit	rps11, rps12 **(2), rps14, rps15, rps16 *, rps18, rps19, rps2, rps3, rps4, rps7(2), rps8
	Subunits of RNA polymerase	rpoA, rpoB, rpoC1 *, rpoC2
	Ribosomal RNAs	*rrn16(2), rrn23(2), rrn4.5(2), rrn5(2)*
	Transfer RNAs	trnA-UGC *(2), trnC-GCA, trnD-GUC, trnE-UUC, trnF-GAA, trnG-GCC *, trnG-UCC, trnH-GUG, trnI-CAU(2), trnI-GAU *(2), trnK-UUU *, trnL-CAA(2), trnL-UAA *, trnL-UAG, trnN-GUU(2), trnP-UGG, trnQ-UUG, trnR-ACG(2), trnR-UCU, trnS-GCU, trnS-GGA, trnS-UGA, trnT-GGU(2), trnT-UGU, trnV-GAC(2), trnV-UAC *, trnW-CCA, trnY-GUA, trnfM-CAU
Other genes	Maturase	*matK*
	Protease	clpP **
	Envelope membrane protein	*cemA*
	Acetyl-CoA carboxylase	*accD*
	c-type cytochrome synthesis gene	*ccsA*
	Translation initiation factor	*infA*
	other	*-*
Genes of unknown function	Conserved hypothetical chloroplast ORF	lhbA, ycf1(2), ycf2(2), ycf3 **, ycf4

Notes: Gene *: gene with one introns; Gene **: gene with two introns; Gene(2): number of copies of multi-copy genes.

## Data Availability

The complete chloroplast genomes generated during the current study were deposited in GenBank with the accession numbers PV191269, OR805597.1, and PP234611~PP233615. The original contributions presented in this study are included in the article/Supplementary Material. Further inquiries can be directed to the corresponding authors.

## Supplementary Materials

The following supporting information can be downloaded at https://www.mdpi.com/article/10.3390/genes16060616/s1, Table S1. Species information and accession numbers of the chloroplast genomes used in the phylogenetic analysis.
